# The Distribution Patterns of Rhesus (Rh) Antigens

**DOI:** 10.7759/cureus.62476

**Published:** 2024-06-16

**Authors:** Babita Raghuwanshi, Kanchan Ahuja, Garima Sharma, Kalpana Sharma, Meghna Singh, Aarti Yadav, Subhash Kumar, Pankaj K Sharma, Harish Chander, Devesh Dubey

**Affiliations:** 1 Transfusion Medicine, All India Institute of Medical Sciences, Bhopal, Bhopal, IND; 2 Blood Reagent Laboratory, National Institute of Biologicals, Noida, IND; 3 Biotherapeutics Research Laboratory, National Institute of Biologicals, Noida, IND

**Keywords:** transfusion, alloimmunization, d antigen, e antigen, rh antigens

## Abstract

Introduction: The determination of one's blood group is dictated by the inheritance-based diversity in the presence or absence of RBC antigens on the surface. Extended Rhesus (Rh) antigens are the most clinically relevant antigens of blood group systems after the ABO blood group system in transfusion medicine. The aim of this study was to serologically assess the prevalence of extended Rh antigens across diverse blood group systems.

Methods: A total of 2043 samples were tested for the ABO blood group and Rh typing with monoclonal antisera. The Rh phenotyping (C, c, E, e ) was performed on all the samples.

Results: The most frequently observed ABO blood group was O (36.5%), while AB (13.6%) was identified as the least prevalent. Positive Rh D antigen was found in 91.6% of tested samples, while 8.4% were Rh D-negative. The most frequently encountered antigen was e, followed by D, while the least prevalent was E.

Discussion: Establishing a Rh phenotype repository for blood donors and conducting Rh phenotype assessments as part of pretransfusion testing before initiating the initial blood transfusion for each patient could significantly lower the patients' incidence of alloimmunization.

## Introduction

The ABO blood group system, pivotal in clinical practice, was first discovered by Karl Landsteiner in the early 1900s. Since its discovery, several blood group antigens have been identified, leading to significant advancements in immunohematology. This progress extends beyond the AB and D antigens, encompassing a spectrum of antigens across various blood group systems, including Rhesus (Rh), Kidd, MNSs, Kell, Lewis, Duffy, Lutheran, and P1PK.

The International Society of Blood Transfusion has recognized 360 red cell antigens and 45 blood group systems [1]. Notably, ABO and Rh (predominantly D) antigens assume paramount importance due to their implications in transfusion and transplantation contexts, predisposing to hemolytic disease in newborns, hemolytic transfusion reactions, and reduced erythrocyte lifespan. Red blood cell antigen phenotyping plays a crucial role in clinical practice, fulfilling various purposes such as identifying alloantibody or autoantibody specificity, matching antigens between donors and recipients, and allocating prophylactic transfusions with matched antigens. This is particularly essential in scenarios involving alloimmunization or autoimmunization.

The principal aim of the blood center is the prompt supply of safe and compatible blood or blood components to patients. Nevertheless, difficulties emerge when dealing with patients with alloantibodies, especially due to the lack of comprehensive donor phenotype databases. Considering the frequent necessity for multiple blood transfusions in the treatment of hematological disorders, pretransfusion testing becomes exceptionally vital The present study aims to serologically assess the prevalence of Rh red cell antigens across diverse blood group systems, encompassing Rh (C, c, E, and e) antigens.

## Materials and methods

The study was carried out from 2016 to 2023 at the Blood Reagent Laboratory of the National Institute of Biologicals, Noida, India, using anonymously stored blood samples from the Indian Red Cross Society Blood Centre, New Delhi, India, in collaboration with the Department of Transfusion Medicine at All India Institute of Medical Sciences, Bhopal, India, a tertiary care multi-specialty hospital. The study protocol was approved by the National Institute of Biologicals Institutional Human Ethics Committee (approval number: NIB/IHEC/2010/01).

Immunohematology testing

A total of 2043 samples were included in the study. All the immunohematological tests in this study were performed with tube agglutination technology (TAT). Samples were tested for the ABO blood group and Rh typing with monoclonal antisera. The Rh phenotyping (C, c, E, e ) was performed on all the samples. Extended phenotyping for all the antigens was performed using reagents procured from Tulip Diagnostics (P) Ltd (Goa, India) and Immucor, Inc. (Georgia, United States). Known positive and negative controls for each antigen were prepared in-house using reagents calibrated against the National Institute for Biological Standards and Control (NIBSC) standards. The antigen phenotyping test procedures were performed per the manufacturer's instructions. Antigens were typed through direct methods, which involved adding antisera to 3-5% of donor red blood cell suspensions or indirectly using an indirect antiglobulin test. Quality controls were ensured by utilizing positive and negative control cells and Coombs’ control cells.

In the context of traditional serological testing using the test tube method, the process involves the identification of antigens by observing the agglutination of red blood cells (RBCs) facilitated by specific antisera, with subsequent assessment of the degree of agglutination. After mixing RBCs with antibodies, the resulting mixture undergoes centrifugation, and the outcome is evaluated through visual inspection. Complete agglutination is indicated if the RBC sediment remains undisturbed after centrifugation and gentle agitation of the test tube. Conversely, non-agglutination is inferred if the RBCs are uniformly suspended throughout the solution.

Result interpretation

The test results thus derived using the TAT were interpreted as follows: the grade of agglutination was noted to range from 4+ to negative; 4+: a solid red agglutinate at the bottom of the tube and no free cells detected, 3+: one or two large agglutinates at the bottom of the tube, 2+: medium-sized agglutinates with a clear background, 1+: small agglutinates with a lot of free red cells, and negative: no agglutination but an even red cell suspension.

The total number of blood samples that tested positive for a particular antigen or phenotype divided by the total number of blood samples tested yielded the prevalence of that particular antigen or phenotype and the results were expressed in percentages.

## Results

A total of 2043 blood samples were tested to determine the frequencies of antigens and phenotypes of the extended Rh blood group system, and D, C, E,c, and e antigens were calculated. The most frequently observed ABO blood group was O (36.5%), while AB (13.6%) was identified as the least prevalent (Table 1).

**Table 1:** Frequencies of ABO blood group antigens

Blood Group	Frequency	Percentage
A	475	23.2
B	546	26.7
AB	279	13.6
O	743	36.5
TOTAL	2043	100.00

Analysis revealed that Rh D positivity was the predominant phenotype, with 91.6% exhibiting the presence of the Rh D antigen on erythrocytes (Table 2). Conversely, 8.4% demonstrated Rh D negativity, indicating the absence of the Rh D antigen on erythrocytes. Among the blood samples analyzed, 1872 tested positive for the Rh D antigen, while 171 were Rh D negative.

**Table 2:** Frequency of Rh D positive and negative in the present study (N =2043) Rh: Rhesus

Rh D Positve, n (%)	Rh D negative, n (%)
1872 (91.6 %)	171 (8.4 %)

All samples were typed for the D, C, E, c, and e antigens in the Rh blood group system analysis. The e antigen exhibited the highest prevalence, observed in 99% of cases (2022 samples), followed by the D, C, and c antigens (91.6%, 84.7%, and 59.5%, respectively) while the E antigen was the least prevalent, found in 20.1% (411 samples) (Table 3). All Rh-negative blood samples had the presence of both c and E antigens in their red cells, as detailed in Table 3. The antigen e was universally present across all samples. These findings highlight the distribution and prevalence of various antigens, shedding light on their immunogenic characteristics.

**Table 3:** The frequency of extended Rh blood group antigens in the present study Rh: Rhesus

Antigen	D		C		c		E		e	
	Frequency	Percentage	Frequency	Percentage	Frequency	Percentage	Frequency	Percentage	Frequency	Percentage
Absent	171	8.4	313	15.3	828	40.5	1632	79.9	21	1.0
Present	1872	91.6	1730	84.7	1215	59.5	411	20.1	2022	99.0

Twelve distinct Rh phenotypes were identified in this study, with the most prevalent being R1 R1 (CDe/CDe), accounting for 40.1% of occurrences, and the rarest being r’r” (CcdEe), found only once. Among the rare Rh phenotypes, R2 RZ, r’r’, and r”r each had a frequency of 0.1%. Among D-positive blood samples, the most common phenotype was R1R1 (40.1%), followed by R1r (30.1%). The frequency of the R2R2 phenotype was 13.4%, while R2r was observed in 5.1% of samples (Table 4).

**Table 4:** Rh phenotype frequency Rh: Rhesus

Phenotype	Frequency	Percentage
R1R1	821	40.1
R1R2	275	13.4
R2r	104	5.1
R1r	615	30.1
R0r	31	1.5
R2R2	150	0.7
R1RZ	8	0.4
rr	152	7.4
r'r	13	0.6
r'r''	1	0.0
r'r'	2	0.1
r''r	2	0.1

## Discussion

In this study, O was the most frequent blood group, followed closely by the B, A, and AB groups. This finding is similar to other studies published in India [2]. The overall global frequency of the B antigen is low, excluding some areas such as central Asia and Africa. In studies from Europe, America, and Southeast Asia, the O antigen is the most frequent blood group [3-7].

In the current study, 2043 samples were phenotyped for extended Rh antigens, which are the most clinically relevant antigens of blood group systems after the ABO blood group system in transfusion medicine. The frequency of the D antigen was high (91.6%) in the present study. However, this frequency is different in the White population, where the prevalence of D is lower at 85% [8]. The e antigen was the most common in our study, similar to other studies [9-10].

R1R1 was the most frequent Rh phenotype identified in the present study as compared to the high prevalence of R1r and Rh-negative phenotype in Caucasians and R0 r in Africans [11-18]. The frequencies of various Rh phenotypes were similar to those found in other Indian studies [2,5,11-15]. Among D-positive donors, R1R1 was the most common, followed by R1r. However, the R1R1 phenotype is present in only 18.5% of White and 2.0% of Black populations [19].

Red cell alloimmunization presents a serious risk to blood transfusion recipients, particularly the group of patients who have received many transfusions. Many factors, including the immunogenicity of the antigen, the frequency of the antigen in the donor population, underlying disease, and genetic factors, might affect the antibody response in a transfused individual [20].

Extensive screening of regular donors for clinically significant antigens is necessary to give multi-transfused patients antigen-negative and antigen-matched blood. Since 60-70% of antibodies are generated against Rh antigens, thalassemia patients' alloimmunization will be significantly reduced if at least some of these antigens are matched. Donors matching by partial matching for Rh antigens (C, c, D, E, and e) will significantly reduce the risk of alloimmunization in multi-transfused patients [21].

In clinical transfusion therapy, using Rh antigen-matched blood represents a fundamental strategy for addressing pertinent challenges. For individuals presenting with the Rh phenotype DCe, it is imperative to administer blood components that have undergone negative screening for Rh antigens E and c, particularly in scenarios where anti-E antibodies are detected during transfusion. This precautionary measure is rooted in the potential immunogenicity induced by anti-E and anti-c predisposing to generating anti-c and anti-cE antibodies, albeit their clinical detection poses challenges. Consequently, the judicious selection of Rh antigen-matched blood components becomes paramount. Analogously, when serological assessments identify the presence of anti-C in patients with the Rh phenotype DcE, opting for blood donations negative for Rh antigens C and e is deemed appropriate.

Different populations' variations in the distribution of Rh phenotypes may result in various alloimmunization incidences. Understanding the distribution of Rh phenotypes within a particular population can then aid in the development of clinical blood transfusion guidelines and help lower significant hemolytic transfusion reactions, particularly delayed hemolytic transfusion reactions (DHTR), difficult donor-recipient matching, lower RBC survival rates following transfusion, and higher blood transfusion needs. Alloimmunization was, in fact, caused mainly by Rh blood group antigens. Dhawan et al. observed that the rate of alloimmunization might reach 5.64%, with Rh blood group antibodies accounting for 52.17% of the cases (anti-E 17%, anti-D 13%, and anti-C 13%) [22].

Nowadays, Rh D typing is common in most nations [23], since the Rh D antigen has a significant immunogenicity in around 80% of Rh D-negative patients [24]. Pathak et al. found that 0.8% of the samples had alloantibodies. The Rh blood group (41.6%) was associated with the highest frequency of antibodies found [25].

Establishing a Rh phenotype repository for blood donors and conducting Rh phenotype assessments as part of pretransfusion testing and before initiating the initial blood transfusion for each patient could potentially facilitate the immediate provision of antigen-negative blood, thereby enhancing the capacity to promptly attend to more patients and reduce the incidence of transfusion reactions. The antigen-matched blood component will significantly lower the patients' incidence of alloimmunization.

Recommendations and limitations

A rare phenotype repository will reduce the lengthy wait period to locate a suitable unit. Therefore, it is necessary to establish the rarity of a blood donor in a given geographic area based on the need for antigen-negative blood transfusion in that specific area, the diversity of the population, and the distribution of antigens. A regional rare donor registry with a data bank of rare phenotype donors can grow with a sizable cohort of donors from that area and eventually contribute to the national rare donor inventory. The knowledge of uncommon antigens and phenotypes in a given geographic area can facilitate blood centers in informing patients and treating physicians of the anticipated wait time for blood.

The study's limitation lies in the fact that the samples are drawn from the urban population of North India, potentially limiting the generalizability of the findings to other populations. Antigen frequency may differ among tribal populations and in other regions of India, highlighting the need for further research to explore these variations comprehensively.

## Conclusions

The Rh phenotyping showed that the most prevalent phenotype was R1R1, occurring in 40.1% of samples, highlighting differences in antigen distribution compared to other populations. These findings underscore the necessity for extensive donor phenotyping, particularly for patients requiring frequent transfusions, to mitigate the risk of alloimmunization and improve transfusion outcomes.

Given the significant variation in Rh antigen distribution among different populations, this study emphasizes the importance of establishing a comprehensive Rh phenotype repository for blood donors. This repository would facilitate the prompt supply of antigen-negative and antigen-matched blood, which is crucial for reducing transfusion reactions and improving patient care. Understanding regional variations in antigen frequency is essential for developing effective transfusion guidelines and improving clinical outcomes for multi-transfused patients across India.
